# Expression and activity of multidrug resistance proteins in mature endothelial cells and their precursors: A challenging correlation

**DOI:** 10.1371/journal.pone.0172371

**Published:** 2017-02-17

**Authors:** Agnieszka Krawczenko, Aleksandra Bielawska-Pohl, Karolina Wojtowicz, Roksana Jura, Maria Paprocka, Elżbieta Wojdat, Urszula Kozłowska, Aleksandra Klimczak, Catherine Grillon, Claudine Kieda, Danuta Duś

**Affiliations:** 1 Hirszfeld Institute of Immunology and Experimental Therapy, Polish Academy of Sciences, Wroclaw, Poland; 2 Department of Histology and Embryology, Poznan University of Medical Sciences, Poznan, Poland; 3 Wroclaw Research Centre EIT^+^, Wroclaw, Poland; 4 Cellular Microenvironment and Pharmacological Targets, Centre de Biophysique Moléculaire, CNRS UPR 4301, Orléans, France; Columbia University, UNITED STATES

## Abstract

Active cellular transporters of harmful agents—multidrug resistance (mdr) proteins—are present in tumor, stem and endothelial cells, among others. While mdr proteins are broadly studied in tumor cells, their role in non-tumor cells and the significance of their action not connected with removal of harmful xenobiotics is less extensively documented. Proper assessment of mdr proteins expression is difficult. Mdr mRNA presence is most often evaluated but that does not necessarily correlate with the protein level. The protein expression itself is difficult to determine; usually cells with mdr overexpression are studied, not cells under physiological conditions, in which a low expression level of mdr protein is often insufficient for detection *in vitro*. Various methods are used to identify mdr mRNA and protein expression, together with functional tests demonstrating their biological drug transporting activities. Data comparing different methods of investigating expression of mdr mRNAs and their corresponding proteins are still scarce. In this article we present the results of a study concerning mdr mRNA and protein expression. Our goal was to search for the best method to investigate the expression level and functional activity of five selected mdr proteins—MDR1, BCRP, MRP1, MRP4 and MRP5—in established *in vitro* cell lines of human endothelial cells (ECs) and their progenitors. Endothelial cells demonstrated mdr presence at the mRNA level, which was not always confirmed at the protein level or in functional tests. Therefore, several different assays had to be applied for evaluation of mdr proteins expression and functions in endothelial cells. Among them functional tests seemed to be the most conclusive, although not very specific.

## Introduction

The endothelium forms a physical, semipermeable barrier that separates blood from surrounding tissues. Under normal, physiological conditions, molecules and circulating substances can be transported across the endothelial barrier directly through endothelial cells or between them [1]. Endothelial barrier integrity differs; in some organs it is more strictly regulated than in others (e.g. blood-brain barrier). Endothelial barrier dysfunction occurs during stimulation by inflammatory agents, pathogens, activated blood cells, or in other disease states [2]. One of the mechanisms maintaining the endothelial barrier function is the activity of transmembrane pumps that could regulate influx and efflux of various substances. The majority of these transmembrane proteins belong to the ABC (ATP-binding cassette) transporters family and some of them are known as multidrug resistance (mdr) proteins. Acting as cellular transporters, ABC proteins participate in normal physiological processes, e.g. secretion in liver hepatocytes and in renal tubule cells [3–4].

ABC transporters are also present in stem and progenitor cells. The mdr proteins expression is connected with stem cells protection from various toxic or harmful molecules. Two of these proteins, expressed in a stem cells subpopulation—BCRP (ABCG2) and MDR1 (ABCB1)—are known as side population determinants [5–6].

Although mdr proteins are extensively studied [7–8], mainly in relation to cancer treatment, methods used for their evaluation are still not satisfactory. The non-functional approach determines the defined mdr mRNA or mdr proteins expression levels. This includes several techniques for RNA and DNA evaluation: reverse transcription PCR (RT-PCR), real-time RT-PCR, Southern and Northern blot, as well as various methods for protein detection: Western blotting, immunofluorescence staining with monoclonal or polyclonal antibodies, or ELISA. However, these methods often give inconsistent results, which can be visible in cells with low mdr proteins expression [9]. Therefore, functional assays are additionally used, based on the ability of mdr proteins to pump fluorescent dyes out of cells.

Detection of mRNA specific for a given protein does not automatically mean that the protein is expressed. There are many mechanisms regulating translation and post-translational processing—among them microRNAs are nowadays extensively described [10].

Another issue is the use of specific antibodies for mdr protein detection. The sensitivity of the method chosen (flow cytometry, Western blotting, ELISA, immunocytochemistry) plays a role, especially when the protein expression level is low. According to the specificity of each technique, the available antibodies are designed to recognize epitopes from denatured proteins up to fully post-translationally modified structures, such as glycoforms [11–12]. Antibody threshold of reactivity is also a determining parameter, especially in dynamic methods of typing, such as flow cytometry. Therefore, protein detected by one method may not be recognized by other method in the same cell sample.

The current study was designed to clarify the question of mdr proteins expression in human endothelial cells and to choose the best method or combination of methods for their evaluation. We compared several methods used for defining the mdr proteins MDR1 (ABCB1), MRP1 (ABCC1), MRP4 (ABCC4), MRP5 (ABCC5) and BCRP (ABCG2), expressed by two unique human endothelial progenitor cell lines—HEPC-CB.1 and HEPC-CB.2 - established by our research group [13], and by endothelial mature cell lines of microvascular HSkMEC.2 [14] and macrovascular (HUVEC) origin. Hence, a model of endothelial cells of different origin and different stage of differentiation—progenitor, derived from microvasculature and from macrovasculature—was proposed. Endothelial progenitors, HEPC-CB.1 and HEPC-CB.2 cells, being at the very early stage of endothelial differentiation, were expected to possess a relatively high expression of mdr protein. In contrast, human microvascular skin endothelial cells HSkMEC.2 represent quiet, mature endothelium and therefore should present a low, “basal” expression level of mdr proteins. HUVEC cells, derived from macrovasculature and having distinct functions, were chosen as a control endothelium.

## Materials and methods

### Reagents

Doxorubicin, rhodamine 123, calcein acetoxymethyl (calcein AM), propidium iodide, verapamil, MK-571 inhibitor and novobiocin were from Sigma Aldrich, USA. Rhodamine 123 and doxorubicin were dissolved in water. All other compounds were dissolved in dimethyl sulfoxide (DMSO, POCh, Poland).

Antibodies used in Western blotting, immunocytochemistry and flow cytometry experiments are listed in Table 1.

**Table 1:** List of antibodies used in the experiments.

		Clone	Recognized epitop	Fluorochrome conjugated	Application	Supplier
Anti-MDR1/ABCB1	MDR1/1	15D3	external	PE	FC	BD Pharmingen
	MDR1/2	17F9	external	PE	FC	BD Pharmingen
	MDR1/3	UIC-2	external		FC	IITD
	MDR1/4	E1Y7S			WB, ICC	Cell Signaling
	MDR1/5	polyclonal			WB	ThermoFisher Scientific
Anti-MRP1/ABCC1	MRP1/1	QCRL-2	internal	FITC	FC	Santa Cruz
	MRP1/2	QCRL-3	internal		FC	IITD
	MRP1/3	polyclonal			WB, ICC	ThermoFisher Scientific
	MRP1/4	polyclonal			WB	Cell Signaling
	MRP1/5	MRPm6			WB	Alexis Biochemicals
Anti-MRP4/ABCC4	MRP4/1	M4I-80			WB, ICC, FC	LifeSpan BioScience
	MRP4/2	D1Z3W			WB, ICC, FC	Cell Signaling
Anti-MRP5/ABCC5	MRP5/1	M5I-10			WB, ICC, FC	Kamiya
Anti-BCRP/ABCG2	BCRP/1	5D3	external	PE	FC	BD Pharmingen
	BCRP/2	BXP-21	internal		WB, ICC, FC	EnzoLife Sciences
	BCRP/3	polyclonal			WB	Cell Signalling
	BCRP/4	BXP-34	internal		WB, ICC	Alexis Biochemicals
Anti-B-actin	B-actin	D6A8			WB	Cell Signaling

FC—flow cytometry, WB—Western blotting, ICC—immunocytochemistry. Antibodies used for FC were unconjugated or directly conjugated to fluorochrome: fluorescein isothiocyanate (FITC) or phycoerythrin (PE). Antibodies MDR1/3 and MRP1/2 were prepared by ourselves at the Institute of Immunology and Experimental Therapy (IITD PAN).

### Cells

Human endothelial progenitor cell lines originated from cord blood (HEPC-CB.1 and HEPC-CB.2) (C. Kieda, Centre National de la Recherche Scientifique, France, European patent N° 1170 3915.6, the USA extended patent N° is 13/521 715) [13] and human normal skin microvascular endothelial cells (HSkMEC.2) (C. Kieda, Centre National de la Recherche Scientifique, France, patent 99–16169) were established according to the method previously described [14]. All these endothelial cells were cultured in Opti-MEM with GlutaMAX (Thermo Fisher Scientific Inc., USA) supplemented with 3% Fetal Bovine Serum (FBS, HyClone, UK) and 1% Penicillin-Streptomycin (Sigma Aldrich, USA) and were routinely passaged using 0.05% trypsin/0.02% EDTA (w/v) solution (IITD PAN, Poland). Human umbilical vein endothelial cells (HUVEC) were isolated from macrovasculature and immortalized with hTERT using a previously described protocol [15]. Cells were cultivated in 199 medium (Lonza, USA) supplemented with 10% FBS (HyClone, UK), L-glutamine (Sigma Aldrich, USA), 1% Penicillin-Streptomycin (Sigma Aldrich, USA) and 200 μg/mL Endothelial Cell Growth Supplement (ECGS, Becton Dickinson, USA). HUVEC cells were cultured on plates coated with collagen (Vitrogen 100, Flow Laboratories Inc., USA).

Human colorectal adenocarcinoma chemoresistant subline LoVo/Dx was obtained by prolonged exposure of LoVo cells (ATCC, USA) to doxorubicin, and was cultured in Ham’s F12 medium (Cytogen, USA), supplemented with 10% FBS (HyClone, UK), L-glutamine (Sigma Aldrich, USA) and 1% Penicillin-Streptomycin (Sigma Aldrich, USA). Doxorubicin (at 100 ng/ml concentration) was constantly present in the culture medium of the LoVo/Dx cells. The drug was withdrawn a week before experiments.

### RT-PCR

Total cellular RNA was isolated from 4 x 10^6^ cells using a NucleoSpin RNA kit (MACHEREY-NAGEL, Germany). First strand cDNA synthesis was performed by reverse transcription of 1 μg of total RNA using the RevertAid First Strand cDNA Synthesis Kit for RT-PCR (Thermo Scientific, USA). The PCR reaction for detecting MDR1, MRP1, MRP4, MRP5 and BCRP mRNAs was performed using specific primers (Table 2). As a control actin mRNA expression was checked. The PCR products were separated on 2% agarose gels and visualized under UV light after ethidium bromide staining. The size of products was estimated using molecular weight marker Gene Ruler 100 bp DNA Ladder (Fermentas, Lithuania). The experiments were repeated at least 3 times.

**Table 2:** Primers used for detection of multidrug resistance proteins.

Primers	Nucleotide sequence	PCR conditions
MDR1	forward: 5’- AAGCTTAGTACCAAAGAGGCTCTG -3’ reversed: 5’- GGCTAGAAACAATAGTGAAAACAA- 3’	[94°C, 1 min; 58°C, 1 min; 72°C, 2 min], 36 cycles Product size: **242 bp**
MRP1	forward: 5’-AGTGACCTCTGGTCCTTAAACAAGG-3’ reversed:5’-GAGGTAGAGAGCAAGGATGACTTGC-3’	[94°C, 30 sec; 58°C, 1 min; 68°C, 1 min], 35 cycles Product size: **657 bp**
BCRP	forward: 5’-CCCAGTACGACTGTGACAATG -3’ reversed: 5’-CACAGTCTTCAAGGAGATCAGCTA -3’	[94°C, 45 sec; 61°C, 30 sec; 72°C, 30 sec], 35 cycles Product size: **135 bp**
MRP4	forward: 5’-TCCTCCTCCATTTACAGTGACA -3’ reversed: 5’-TTATTCTCCTAAACACTGCAGCTC-3’	[94°C, 45 sec; 61°C, 30 sec; 72°C, 30 sec], 35 cycles Product size: **110 bp**
MRP5	forward: 5’-TGAATCTGAAGTGATGGAGAATGG -3’ reversed: 5’-CCTATCGGAGCCTAGAACCG -3’	[95°C, 45 sec; 52°C, 1 min; 72°C, 1 min], 35 cycles Product size: **232 bp**
Actin	forward: 5’- CCAGAGCAAGAGAGGCATCC-3’ reversed: 5’- CTGTGGTGGTGAAGCTGAAG-3’	[95°C, 30 sec; 57°C, 30 sec; 72°C, 30 sec], 30 cycles Product size: **450 bp**

### Western blotting

Endothelial cells were plated on Petri dishes (6 cm) and incubated in standard cell culture conditions for 24 h. Cells were then scraped and lysed in RIPA lysis and extraction buffer (Thermo Scientific, USA) and kept at -80°C. The protein content in cell extracts was determined using BCA Protein Assay Kit (Pierce^™^ Thermo Scientific, USA). 50 μg of total protein per lane was subjected to SDS-PAGE and transferred onto Immobilon PVDF Membrane (Merck Millipore, Germany). The membrane was blocked with a 5% solution of BLOT-QuickBlocker^™^ (Calbiochem, USA) for 1 h at room temperature. Further incubations were performed in PBS containing 1% BLOT-QuickBlocker^™^. Protein levels were determined with specific antibodies against: MDR1 (anti-MDR1/4, anti-MDR1/5); MRP1 (anti-MRP1/3, anti-MRP1/4 and anti-MRP1/5); MRP4 (anti-MRP4/1 and anti-MRP4/2); MRP5 (anti-MRP5/1); BCRP (anti-BCRP/2, anti-BCRP/3 and anti-BCRP/4); and β actin. The membrane was incubated with the specific antibody for 1 h at room temperature. After washing three times with 0.05% (v/v) Tween-20 solution in PBS, the membrane was incubated with secondary biotinylated antibody (Dako, USA) for 1 h and washed three times with 0.05% Tween-20 in PBS. Finally, the membrane was incubated with streptavidin-HRP (Dako, USA). Chemiluminescent reaction was developed using ECL Western Blotting Substrate (Promega, USA) and visualized on CL-XPosure film (ThermoFisher Scientific, USA). As a positive control human LoVo/Dx cell lysate was used. The experiments were repeated at least 3 times. The concentrations of antibodies are presented in Table 3.

**Table 3:** Antibody concentrations used for Western blotting analysis of multidrug resistance protein expressed by endothelial cells.

	Anti-β actin	Anti-MDR1		Anti-MRP1			Anti-MRP4		Anti-MRP5	Anti-BCRP		
1st Ab	β actin	MDR1/4	MDR1/5	MRP1/3	MRP1/4	MRP1/5	MRP4/1	MRP4/2	MRP5/1	BCRP/2	BCRP/3	BCRP/4
	1:100	1:1000	1:1000	1:2000	1:1000	1:180	1:100	1:100	1:100	1:1000	1:1000	1:500
2nd Ab	Goat anti-rabbit biotinylated	Goat anti-rabbit biotinylated	Goat anti-rabbit biotinylated	Goat anti-rabbit biotinylated	Goat anti-rabbit biotinylated	Goat anti-rat biotinylated	Goat anti-rat biotinylated	Goat anti-rabbit biotinylated	Goat anti-rat biotinylated	Goat anti-mouse biotinylated	Goat anti-rabbit biotinylated	Goat anti-mouse biotinylated
	1:4000	1:4000	1:4000	1:4000	1:4000	1:2860	1:1000	1:4000	1:1000	1:10000	1:4000	1:10000
3rd Ab	Streptavidin/HRP	Streptavidin/HRP	Streptavidin/HRP	Streptavidin/HRP	Streptavidin/HRP	Streptavidin/HRP	Streptavidin/HRP	Streptavidin/HRP	Streptavidin/HRP	Streptavidin/HRP	Streptavidin/HRP	Streptavidin/HRP
	1:50000	1:50000	1:50000	1:50000	1:50000	1:2500	1:4000	1:50000	1:4000	1:50000	1:50000	1:5000

### Flow cytometry—Protein analysis

For MRP1 and BCRP (BXP-21 clone) staining cells were permeabilized using the Fixation/Permeabilization Solution Kit (BD Biosciences, USA). For other stainings cells were detached using NonEnzymatic Cell Dissociation Solution (ATCC, USA). Next all cells were labeled with antibodies specific for CD243 (anti-MDR1/1, anti-MDR1/2 and anti-MDR1/3); MRP1 (anti-MRP1/1 and anti-MRP1/2); MRP4 (anti-MRP4/1 and anti-MRP4/2); MRP5 (anti-MRP5/1); BCRP (anti-BCRP/1 and anti-BCRP/2) and the appropriate isotypic control for 30 min at 4°C (Table 4).

**Table 4:** List of control antibodies used in flow cytometry experiments.

		Fluorochrome conjugated	Supplier
1 st Ab	Mouse IgG1	PE	BD
	Mouse IgG2b	PE	BD
	Mouse IgG2a		R&D Systems
	Mouse IgG2b	FITC	BD
	Mouse IgG1		R&D Systems
	Rabbit IgG		ThermoFisher Scientific
	Rat IgG2a		BD
2nd Ab	Goat anti-Rat IgG	Alexa Fluor^®^ 488	ThermoFisher Scientific
	Goat anti-Mouse IgG	FITC	Sigma
	Goat anti-Rabbit IgG	FITC	Sigma

After washing with PBS cells were analyzed or detection by incubation with the corresponding FITC-labeled secondary antibody (Sigma Aldrich, USA) was performed for an additional 30 min at 4°C. After washing with PBS cells were analyzed using a FACSCalibur flow cytometer, and data were processed using CellQuest software (BD Biosciences, USA) for 3 independent experiments. MDR protein expression was evaluated using the Kolmogorov-Smirnov statistic automatically calculated by CellQuest software (D value ≥0.2 was evaluated as positive). The concentrations of used antibodies are presented in Table 5.

**Table 5:** Antibody concentrations used for flow cytometry analysis of multidrug resistance proteins expressed by endothelial cells.

	Anti-MDR1			Anti-MRP1		Anti-MRP4		Anti- MRP5	Anti -BCRP	
1 st Ab	MDR1/1	MDR1/2	MDR1/3	MRP1/1	MRP1/2	MRP4/1	MRP4/2	MRP5/1	BCRP/1	BCRP/2
	1:50	1:50	1:5000	1:50	1:10000	1:100	1:100	1:100	1:50	1:100
2nd Ab			FITC anti-mouse		FITC anti-mouse	Alexa488 anti-rat	FITC anti-rabbit	Alexa488 anti-rat		FITC anti-mouse
			1:200		1:200	1:200	1:200	1:200		1:200

### Flow cytometry—Functional test

#### A) Standard functional test

Endothelial cells or LoVo/Dx cells were incubated with the appropriate concentration of rhodamine 123 or calcein AM (final concentration 0.2 μM and 0.05 μM, respectively) for 60 min at 37°C. After washing once in cold medium, cells were incubated for 2 h at 20°C in growth medium or growth medium with inhibitors specific for three major ABC transporter types: 10 μM verapamil (MDR1 inhibitor), 25 μM MK-571 (MRP inhibitor) or 20 μM novobiocin (BCRP inhibitor). Inhibitor concentrations were chosen based on previously published data, demonstrating their lack of toxicity towards endothelial cells, even after long incubation period [16–17]. However, MDR1 and BCRP inhibitors concentrations used in standard tests were reduced by half, as they were cytotoxic for endothelial cells after 2h incubation at 20°C. Moreover, MK-571 inhibitor was found to be toxic for endothelial cell lines tested; therefore its concentration was reduced from 50 μM to 25 μM in both functional tests. After incubation with inhibitors cells were placed on ice and propidium iodide (2.5 μg/mL) was added before data acquisition. Cell fluorescence was measured by flow cytometry using a Becton Dickinson FACSCalibur analyzer equipped with a 488 nm argon laser. Fluorescence was recorded via a 530/30 nm band pass filter. A total of 5,000 alive (propidium iodide negative) cells were registered as events and analyzed using Cell Quest software. The influence of DMSO (maximal concentration in samples 0.8%) on cell viability was also monitored. Multidrug resistant protein Activity Factor (MAF) was calculated from the following equation on the basis of measured mean fluorescence intensity values (MFI): MAF [%] = 100 × ((MFI inhib—MFI med)/MFI inhib)), where MFI inhib is the MFI value for cells incubated in the presence of a specific inhibitor, while MFI med is the MFI value for cells incubated in medium with DMSO [18–19].

#### B) Commercial functional test

EFluxx-ID Green Multidrug Resistance Assay Kit purchased from Enzo Life Sciences company (USA) was used according to the manufacturer’s instructions. The recommended concentration of MRP inhibitor was reduced to 25 μM because of cytotoxicity for endothelial cells. Before starting the test all cells were kept in complete growth medium without Phenol Red (OptiMEM with GlutaMAX, Thermo Fisher Scientific Inc., USA).

The substrates and inhibitors used in functional tests are presented in Table 6.

**Table 6:** Substrates and inhibitors used in functional tests.

	Protein	Substrate	Inhibitor
Standard test	MDR1	rhodamine 123	verapamil
	MRP	calcein AM	MK-571
	BCRP	rhodamine 123	novobiocin
Commercial test	MDR1	eFluxx-ID^**®**^ Green	verapamil
	MRP	eFluxx-ID^**®**^ Green	MK-571
	BCRP	eFluxx-ID^**®**^ Green	novobiocin

In both functional tests (standard and commercial) cells with MAF values <25% should be regarded as multidrug resistance negative. For each experiment all three MAF values were used to calculate the mean MAF value. The differences between sets of measurements were below 10%. All the experiments were repeated at least 3 times.

### Immunocytochemistry

#### Cytospin preparation

Cell lines HEPC-CB.1, HEPC-CB.2, HSkMEC.2, HUVEC, and LoVo/Dx were cultured for 3 days, then trypsinized and resuspended in PBS with final density of 1 x 10^6^ cells/ml. Cytospin slides were prepared and dried overnight at room temperature and then stored at -20°C until immunostaining.

#### Immunostaining

To assess the expression of mdr proteins in the analyzed cells, cytospin slides were thawed at room temperature and then fixed in acetone for 10 min. Then slides were left for 20 min.—until complete solvent evaporation—and placed in washing buffer TRIS/NaCl pH 7.6 for 5 min. Monoclonal antibodies were diluted in the antibody diluent (Dako, Glostrup, Denmark) as follows: anti-MDR1/4 (1:400), anti-MRP1/3 (1:100), anti-MRP4/2 (1:100), anti-MRP5/1 (1:100), anti-BCRP/2 (1:100). Then they were incubated with cells for 1h at room temperature. Between each step of immunostaining slides were washed in TRIS/NaCl buffer pH = 7.6. Visualization was performed by using the Dako EnVision G/2 System/AP kit, detecting mouse and rabbit primary antibodies. Slides were counterstained with Mayer’s Hematoxylin (Bio-Optica, USA), washed in distillated water, and then mounted with Faramount medium (Dako, Glostrup, Denmark). Slides incubated with an appropriate secondary antibody (rabbit/mouse LINK, Dako) served as a negative control. The LoVo/Dx cell line served as a positive control. Protein expression was assessed by staining intensity. Intensity of staining was scored subjectively as follows: no staining (-), weak (+), moderate (++) and strong (+++). Slides were analyzed and images were recorded using an Axioplan 2 (Zeiss, Jena, Germany) microscope under magnification 200x. Staining was repeated at least 3 times for each mdr protein tested.

## Results

### Mdr protein mRNA expression

**Fig 1:**
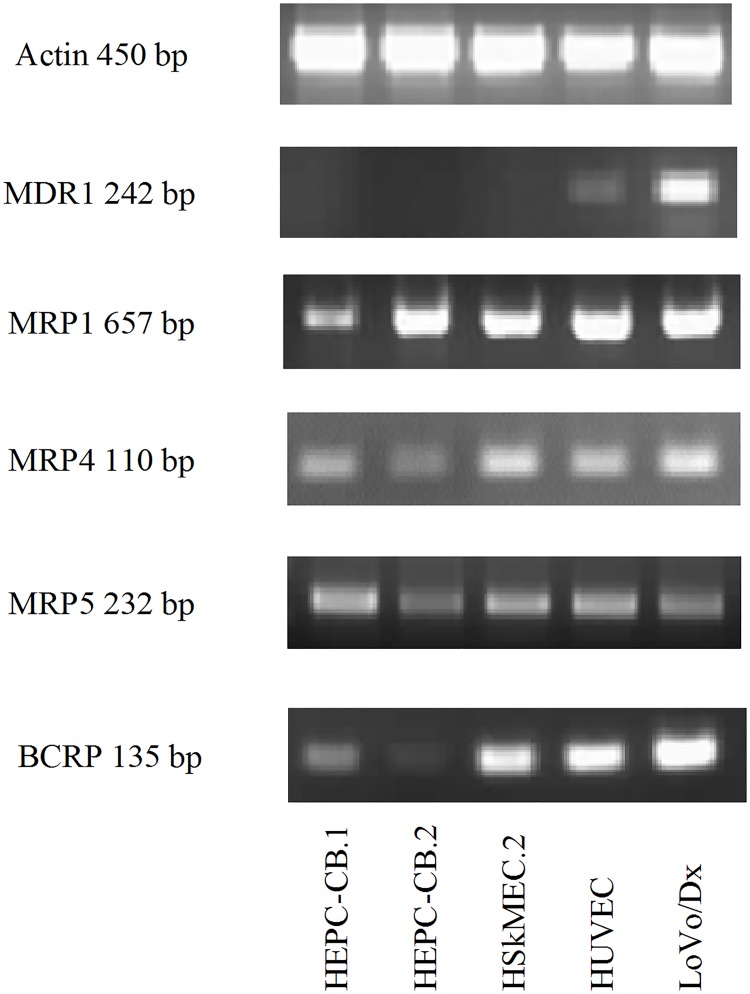
Expression of mdr proteins mRNA in endothelial cells using RT- PCR method. The primers used for the reaction are listed in Table 2. As a control actin mRNA expression was checked. LoVo/Dx cells served as a positive control.

The mRNAs for the mdr proteins: MDR1, MRP1, MRP4, MRP5 and BCRP were detected using reverse transcription PCR (RT-PCR). The mRNAs for those proteins were found in all endothelial cells tested, except MDR1 mRNA, which was found only in HUVEC cells (Fig 1). Tumor LoVo/Dx cells were used as a positive control cells in all methods.

### Mdr detection by flow cytometry

The mdr detection at the protein level turned out to be a challenge. The first method used was flow cytometry. Monoclonal antibodies used originated from three distinct clones recognizing MDR1, two clones recognizing MRP1, MRP4 or BCRP, and one clone recognizing MRP5. Results are presented in Table 7. Representative histograms are shown in S1 Fig and raw data are presented in S1 File (see supplementary material). None of the investigated endothelial cell lines was MDR1 positive, whereas MRP1 expression evaluation depended on the antibody clone used. Cells labeled with clone QCRL-2 (anti-MRP1/1) did not reveal MRP1 protein expression, whereas cells stained with clone QCRL-3 (anti-MRP1/2) showed a significant MRP1 protein expression level. For MRP4 expression HEPC-CB.1, HEPC-CB.2 and HSkMEC.2 cells were found positive after treatment with two different clones: anti-MRP4/1 and anti-MRP4/2. HUVEC cells were found negative with anti-MRP4/1 but positive with anti-MRP4/2 antibodies. All endothelial cell lines tested were MRP5 protein positive, as revealed with only one antibody used. Endothelial progenitor cells showed BCRP protein expression only when anti-BCRP/2 clone was used; with anti-BCRP/1 clone no reaction was observed. Mature endothelial cells, both HSkMEC.2 and HUVEC, were BCRP negative regardless of the antibody clone used. LoVo/Dx cells always showed positive staining, except for anti-MRP4/2 clone.

**Table 7:** Flow cytometry analysis of multidrug resistance protein expressed by endothelial cells.

	Anti- MDR1			Anti-MRP1		Anti-MRP4		Anti-MRP5	Anti-BCRP	
	MDR1/1	MDR1/2	MDR1/3	MRP1/1	MRP1/2	MRP4/1	MRP4/2	MRP5	BCRP/1	BCRP/2
HEPC-CB.1	D = 0.02	D = 0.02	D = 0.02	D = 0.00	**D = 0.59**	**D = 0.51**	**D = 0.38**	**D = 0.58**	D = 0.00	**D = 0.59**
HEPC-CB.2	D = 0.02	D = 0.02	D = 0.02	D = 0.00	**D = 0.63**	**D = 0.32**	**D = 0.20**	**D = 0.29**	D = 0.00	**D = 0.25**
HSkMEC.2	D = 0.02	D = 0.02	D = 0.02	D = 0.00	**D = 0.52**	**D = 0.20**	**D = 0.36**	**D = 0.63**	D = 0.00	D = 0.00
HUVEC	D = 0.02	D = 0.02	D = 0.02	D = 0.00	**D = 0.60**	D = 0.02	**D = 0.49**	**D = 0.44**	D = 0.00	D = 0.00
LoVo/Dx	**D = 0.94**	**D = 0.52**	**D = 0.87**	**D = 0.51**	**D = 0.94**	**D = 0.81**	D = 0.17	**D = 0.26**	**D = 0.81**	**D = 0.75**

Protein expression was evaluated using specific antibodies or isotype control. Results are shown as the D value using the Kolmogorov-Smirnov statistic for one representative experiment. *D* values ≥0.20 were evaluated as positive.

### Mdr detection by Western blotting

**Fig 2:**
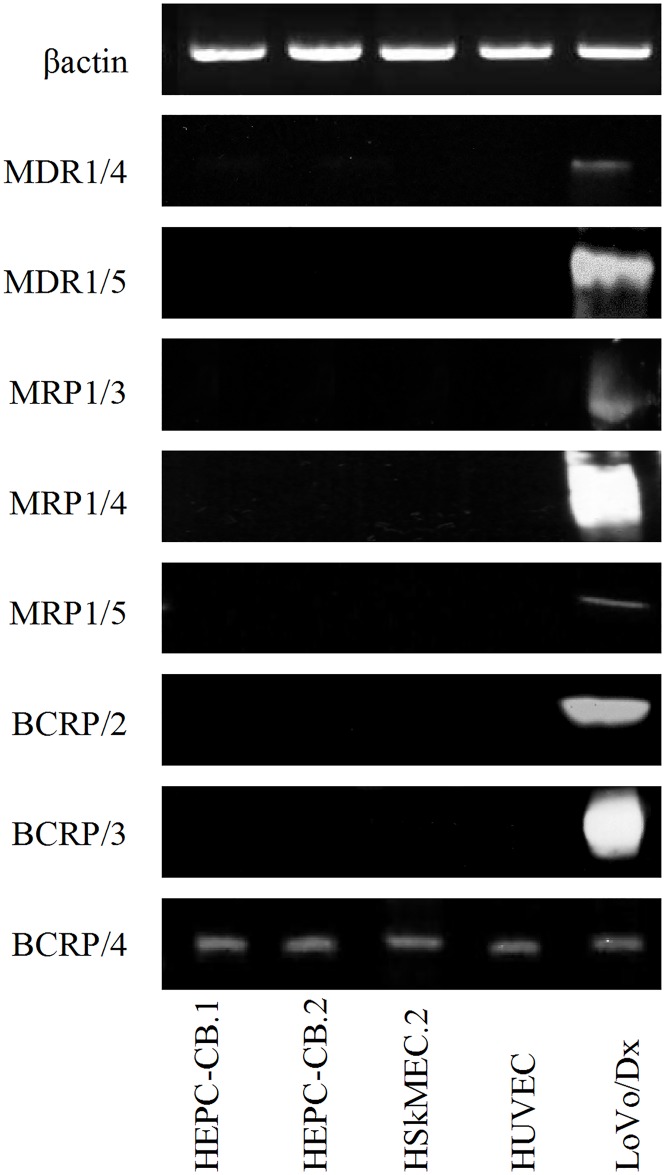
Western blotting analysis of multidrug resistance protein in endothelial cells. MDR1, MRP1 and BCRP protein levels were revealed with different sets of specific antibodies in endothelial cell extracts obtained lysing cells with RIPA buffer. Protein extracts from LoVo/Dx cells were analyzed as positive controls. MDR1/4: 180 kDa; MDR1/5: 170 kDa, MRP1/3: 180 kDa; MRP1/4: 170–220 kDa; MRP1/5: 190 kDa; BCRP/2: 72 kDa; BCRP/3 65–80 kDa and BCRP/4: 72 kDa. Equal protein loading (50 μg/line) was confirmed by β-actin expression (45 kDa).

To further decipher the mode of mdr expression, Western blotting was applied (Fig 2). Antibodies recognizing MDR1: anti-MDR1/4 and anti-MDR1/5 reacted only with control LoVo/Dx tumor cells, which confirmed the negative results obtained for expression of their mRNAs. The opposite situation was observed for MRP1 protein: a positive reaction was found only in LoVo/Dx cells, even though all endothelial cells tested expressed MRP1 mRNA. BCRP detection depended on the antibody clone used in the experiment. The anti-BCRP/4 antibody gave positive staining for all cells examined, whereas anti-BCRP/2 antibody gave no positive reaction, as shown in Fig 2. With anti-MRP4/1, anti-MRP4/2 and anti-MRP5/1 antibodies we did not observe any positive reaction, even with LoVo/Dx control tumor cells [data not shown].

### Mdr expression evaluated by immunocytochemistry

**Fig 3:**
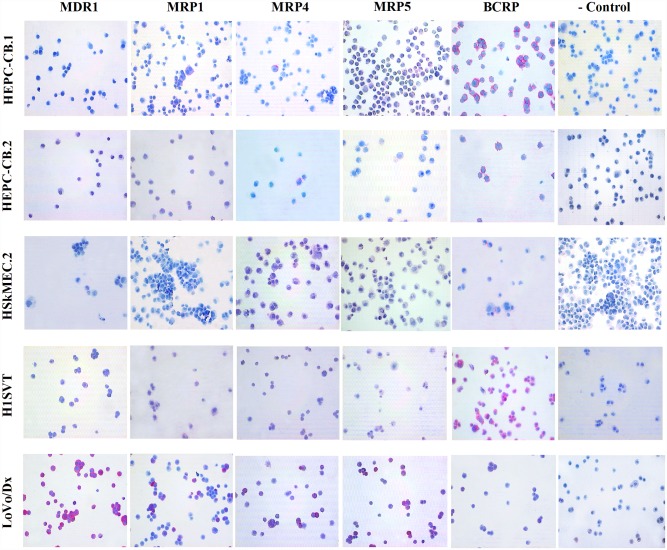
Immunocytochemical staining of endothelial cell lines on cytospin slides for mdr proteins. LoVo/Dx cell line was used as a positive control. Slides incubated with secondary antibody (rabbit/mouse link) served as a negative control.

The next step was immunocytochemical method application. Positive control LoVo/Dx cells expressed all examined proteins including MDR1, MRP1, MRP4, MRP5 and BCRP, although with diverse intensity. Strong staining was observed for MDR1, MRP1, MRP4, and MRP5, whereas BCRP expression was found to be faint (Fig 3). For endothelial cells MDR1 expression was not observed. Single cells expressing MRP1 or MRP4 were found among HEPC-CB.1 and HEPC-CB.2 cells. Weak or no expression of MRP1 and MRP4 was observed for HSkMEC.2 and HUVEC cells. Weak expression of MRP5 was present only in HEPC-CB.1 cells, while other endothelial cell lines were found negative. BCRP staining revealed high expression in HEPC-CB.1 and HUVEC cells, moderate expression in HEPC-CB.2 cells, and weak in HSkMEC.2 cells (Fig 3).

The results of Western blotting and immunocytochemistry for MDR1 and BCRP using anti-BCRP/4 antibody staining were in accordance with their mRNA expression, whereas we did not observe such a correlation for MRP proteins. Therefore, the functional activities of mdr proteins were tested.

### Functional assessment of mdr proteins

**Fig 4:**
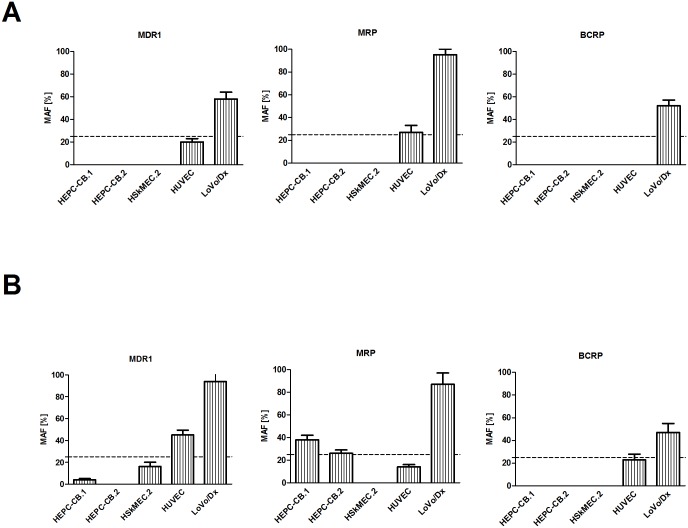
Comparison of MDR activity factor (MAF) of endothelial cells: A) standard functional test; B) commercial functional eFluxx-ID Green test. **A)** Cell lines were trypsinized, washed with medium and incubated with rhodamine 123 or calcein AM dyes. After one wash, cells were incubated in medium or medium with specific inhibitors: 10 μM of verapamil, 25 μM of MK-571 or 20 μM of novobiocin. **B)** Cell lines were trypsinized, washed with PBS, aliquoted and treated in triplicate with different inhibitors (20 μM of verapamil, 25 μM of MK-571, or 50 μM of novobiocin) or untreated (medium with DMSO). Tested probes (eFluxx-ID Green) were added to every sample apart from one tube (white cells). The cells were incubated with the dye in the presence or absence of inhibitors for 30 min. at 37°C.

For investigation of mdr proteins activities, two functional tests were applied. In the standard test, cells were incubated with fluorescent dye (rhodamine 123 or calcein AM) and, after incubation, retention of the dye in the presence of specific inhibitors was measured (Fig 4A). The second test used was the commercially available eFluxx-ID^®^ Green Multidrug Resistance Assay Kit (Fig 4B, commercial test). Multidrug resistance protein activities were observed in HEPC-CB.1, HEPC-CB.2 and HUVEC cells. In the standard functional test HUVEC cells showed low MRP activity (Fig 4A). When the commercial test was applied, only the activity of the MDR1 pump was observed for HUVEC cells and MRP activity for HEPC-CB.1 and HEPC-CB.2 cells (Fig 4B). Representative histograms are shown in S2 Fig and raw data are presented in S1 File (see supplementary material). Positive control LoVo/Dx cells confirmed activities of all ABC pumps tested. The differences between the results of these two functional tests may be due to higher sensitivity of commercial probes of e-Fluxx-ID Green Kit as compared to other mdr substrates. Moreover, the commercial probe was the substrate for all mdr proteins investigated, as compared to two different probes used in the standard test (rhodamine 123 and calcein AM).

Then cells were immediately analyzed by flow cytometry. The representative MAF index from at least three independent experiments was shown. MAF>25% is regarded as multidrug resistance positive (red line is MAF = 25%).

Results obtained in all experiments are summarized in Table 8.

**Table 8:** Summary of results. Numbers indicate positive (1) or negative (0) results obtained by particular technique used. In case of ICC the gradation of results is presented as (+) and (-) score. M- mRNA; WB- Western Blotting FC- Flow Cytometry; ICC- immunocytochemistry; FA- standard functional test; FB- commercial functional test. FA* and FB* refers to functional tests applied for the whole MRP protein family. All tests were repeated at least three times, and both functional tests were each time performed in triplicate.

	MDR1						MRP1						MRP4				MRP5				BCRP					
	M	WB	FC	ICC	FA	FB	M	WB	FC	ICC	FA*	FB*	M	WB	FC	ICC	M	WB	FC	ICC	M	WB	FC	ICC	FA	FB
HEPC-CB.1	0	00	000	-	0	0	**1**	000	0**1**	+	0	**1**	**1**	0	**11**	+	**1**	0	**1**	-/+	**1**	00**1**	0**1**	+++	0	0
HEPC-CB.2	0	00	000	-	0	0	**1**	000	0**1**	+	0	**1**	**1**	0	**11**	+	**1**	0	**1**	-	**1**	00**1**	0**1**	++	0	0
HSkMEC.2	0	00	000	-	0	0	**1**	000	0**1**	-	0	0	**1**	0	**11**	-/+	**1**	0	**1**	-	**1**	00**1**	00	-/+	0	0
HUVEC	**1**	00	000	-	0	**1**	**1**	000	0**1**	-	**1**	0	**1**	0	0**1**	-	**1**	0	**1**	-	**1**	00**1**	00	+++	0	0
LoVo/Dx	**1**	**11**	**111**	+++	**1**	**1**	**1**	**111**	**11**	+++	**1**	**1**	**1**	0	**1**0	+++	**1**	0	**1**	+++	**1**	**111**	**11**	++	**1**	**1**

## Discussion

In most previous reports mdr proteins were investigated in human tumor cells, as being associated with frequent cancer treatment failure. ABC transporter expression was further reported in several other cell types, including blood-brain barrier endothelial cells, liver and kidney [3, 20–21].

Based on the observation that endothelial cells isolated from brain vessels express mdr proteins [22–23], we performed these studies to investigate their expression on endothelial cells of different tissue origin and at different levels of differentiation. Multidrug resistance protein gene expression is often analyzed at the mRNA level, using RT-PCR and real-time RT-PCR methods, due to their sensitivity [21, 24]. We found mRNA for several mdr proteins detectable in endothelial cell progenitors as well as in mature HSkMEC.2 and HUVEC endothelial cells. Our results are in good accordance with a previously reported experiment testing human mature endothelial cells [25]. However, mRNA presence does not always reflect the final protein expression and transporter functions [26–27]. Some researchers have even reported that tumor cells with high mdr mRNA expression levels did not express them at the protein level [24, 28]. Therefore, studies with the application of specific antibodies against particular mdr proteins were conducted.

Many different antibodies, monoclonal and polyclonal, are used for the measurement of mdr proteins’ presence using several immunochemical protocols, such as flow cytometry, Western blotting or immunocytochemistry. Flow cytometry turned out to be the preferable method for mdr assessment due to its sensitivity and simplicity. We tested three different antibody clones for MDR1 protein, two clones for MRP1, two clones for MRP4 and two clones for BCRP. For all antibodies applied, except for anti-MRP4/2 antibody, positive reactions were observed with LoVo/Dx positive control cells. In endothelial cell lines diverse antibody clones gave varying results. Even antibodies against one specific mdr protein may recognize different epitopes, and their detection sensitivity may differ. So, antibodies designed towards one specific determinant may provide dissimilar results. The most frequently chosen antibodies are those recognizing extracellular epitopes of mdr protein, and being directly conjugated with fluorochrome [29]. In this study we used anti-BCRP/1 antibody recognizing an external epitope, but it gave negative results with both HEPC-CB.1 and HEPC-CB.2 cell lines, whereas anti-BCRP/2 antibody, which recognizes an internal epitope, gave positive results (see Table 7). Using positive and negative controls and excluding the methodical difficulties, we noticed that antibodies are not always suitable reagents. Insufficient specificity, sensitivity and lot-to-lot consistency may generate false results and unnecessary expenses. Similar observations were reported by Baker [16] and Kosztyu et al. [24].

For Western blotting analysis of mdr proteins expression we observed an analogous situation. At least two different antibodies specific for one mdr protein were tested. Different clones of specific antibodies gave divergent results. We found the expression of MDR1, MRP1 and BCRP proteins in LoVo/Dx positive control cells. For MDR1 staining neither anti-MDR1/4 nor anti-MDR1/5 antibodies were able to detect MDR1 protein expression in endothelial cells (Fig 2), which confirmed the lack of mRNA expression for MDR1 found by us in all endothelial cells tested. Endothelial cells, both progenitor and mature HSkMEC.2 and HUVEC, revealed BCRP presence only when anti-BCRP/4 antibody was used; no such presence BCRP was found using anti-BCRP/2 antibody, which was opposite to the results of cytometric analysis of endothelial progenitor cells. According to commercial notes, both antibodies—anti-BCRP/2 and anti-BCRP/4—recognize an internal epitope, and the lack of staining by anti-BCRP/2 in Western blotting was an unexpected observation. MRP proteins MRP1, MRP4 and MRP5 were not detected by Western blotting, and again this was in contrast to the results obtained by flow cytometry. In both methods, Western blotting and flow cytometry, the same clones of antibodies against MRP4 and MRP5 were used. The only difference found was associated with MRP1 detection; flow cytometric measurements were done with anti-MRP1/1 and anti-MRP1/2 antibodies, whereas in Western blotting anti-MRP1/3, anti-MRP1/4 and anti-MRP1/5 were used. However, MPR4 and MRP5 protein expression was not found, even in the positive LoVo/Dx control cells.

In all endothelial cells tested with the immunocytochemical method, MDR1 protein expression was not detectable, whereas BCRP expression was visible, which does not match the results obtained either by flow cytometry or in Western blotting, using the same anti-BCRP/2 antibody clone. Similar confusing results were found for MRP protein expression. For HEPC-CB.1 and HEPC-CB.2 cell lines only single cells with MRP1 and MRP4 expression were observed. MDR protein expression was demonstrated to be related to cell cycle phase [30]. As a consequence, it is possible that endothelial progenitor cells fixed in different cell cycle phases during cytospin preparation were differently labeled. Similar situation is observed when cells are blocked in a specific cell cycle phase after treatment with chemotherapeutic agents. High mdr protein expression level in this phase may induce chemoresistance [31–32]. LoVo/Dx control cells were found to be positive for all mdr proteins tested by immunocytochemistry.

The possibility that endothelial cells treated with chemotherapeutics, arrested in a defined cell cycle phase, may induce their mdr protein expression, needs to be examined. However, all the experiments performed in our laboratory showed that chemotherapeutics exhibit strong cytotoxicity towards endothelial cells (data not shown).

Our previous research has been focused on the expression of mdr proteins in acute myeloid leukemia human blasts. We found that MDR1 protein overexpression, and co-expression of other mdr proteins at diagnosis, are the factors associated with treatment failure in acute myeloid leukemia patients [33]. However, these studies were performed on tumor cells with relatively high mdr protein expression, as compared to normal endothelial cells. The mdr protein expression on tumor cells is intended to protect the tumor from toxic substances, whereas the endothelium seems to be much more sensitive to different toxic factors. Therefore, drug stimulation of endothelial cells to induce mdr protein expression is a challenging aspect.

One should also remember that expression of mdr proteins does not always correlate with their mRNA levels [9, 34]. Such a positive correlation was observed only for positive control tumor cell lines, where the number of protein transcripts is present at the detection level. Measurable mdr protein expression in these cells is often induced by specific drug treatment, whereas normal non-tumor cell lines demonstrate only low mdr expression. On the other hand, the mdr protein expression level—measured by classical methods—do not correlate with their functional activities [24, 34]. Protein expression data only roughly reflect their transporter functions, as these can be modulated by various factors [35]. Another aspect is the fact that the difficulties regarding functional test results are most often connected with the specificity/selectivity of substrates and inhibitors used in this technique. Many commonly used florescent substrates such as calcein AM and rhodamine 123 are not selective and may be removed from the cell by two or more mdr transporters [36–39]. Therefore, there is a need for application of selective inhibitors, and this approach was applied in the presented experiments. This is also a general principle of the commercial eFluxx-ID Green Multidrug Resistance Assay Kit, where the same substrate for all measured mdr proteins is used and particular mdr protein activity is distinguished by using different selective inhibitors. Our results show that the commercially available functional test eFluxx-ID Green Multidrug Resistance Assay Kit is more sensitive than the standard one. Using the commercially available test we observed the MDR1 protein activity in HUVEC cells and MRP activity in HEPC-CB.1 and HEPC-CB.2 cells. Only for positive control LoVo/Dx cells did we fail to observe differences in mdr protein activities revealed by both functional tests. One of the main reasons is that the mdr protein expression level—detectable in LoVo/Dx cells using both methods mentioned above—is sufficient for inducing their transporting activity. It was previously shown that mdr protein expression might be induced on the surface of the tumor cells after a specific drug treatment [40–41]. Nevertheless, ABC transporter expression could not be induced in endothelial cells investigated in our laboratory, according to previously published protocols [42]. The endothelial cells isolated from skin, and cultured in the presence of doxorubicin died, and we did not succeed in obtaining a doxorubicin resistant subline, even at the lowest doxorubicin concentration used. It is noteworthy that endothelial cells are very sensitive to chemical agents; in standard functional tests we had to reduce the recommended inhibitor concentrations, as they were highly cytotoxic for endothelial cells after the proper incubation time.

Our results strongly suggest that it is quite difficult to establish correlations between mdr mRNAs and presence of a given protein or its functional activities; therefore, sets of different methods should be applied for evaluation of mdr expression levels and for investigation of their biological activities. From the clinical point of view the most important may be mdr protein functions; therefore a functional test (standard or commercial) should be conclusive. Functional assays may offer an advantage over antigen measurements since they measure the real mdr protein transport activities [43]. However, it should not be forgotten that in many cells, even in the primary absence of mdr protein activities, they may be further induced by chemical agents. Moreover, in the case of MRP transporters these tests are able to measure the general MRP pumps’ activity only. Consequently, in order to test the accurate MRP protein expression, e.g. MRP1 or MRP4, one needs to apply specific antibodies for evaluation of their presence in tested cells.

Additionally, epitope density should be also taken into account. Our results indicate that positive LoVo/Dx cells express mdr proteins which were functionally active in all experiments performed. The situation was not so clear concerning endothelial cells. Mdr proteins’ epitope densities were too low to be detected by Western blotting; different results were obtained for flow cytometry and immunocytochemistry staining. This may also be related to antibodies’ way of action. Antibodies recognize three-dimensional structures and antigen conformation may be quite different regarding the method of its detection. In flow cytometry and immunocytochemistry the protein is nearer its native form than in Western blotting where heating and SDS usage strongly change the antigen conformation. Therefore, it is possible that some epitopes detected by the immunocytochemistry method are not recognized in Western blotting. On the other hand, antibodies are most often raised to peptide sequences and hence bind better to peptide chains than their native conformation. In Western blotting the protein is often denatured; so peptide chains are available for the antibody to bind. Therefore, one should carefully choose antibodies designed to work in the selected technique of measurement. This explanation, however, cannot be applied to the results obtained by our group for BCRP protein investigated in endothelial cells by immunocytochemistry and Western blotting. Both clones of antibody used, BXP-21 (BCRP/2) and BXP-34 (BCRP/4), recognize an internal epitope and are designed for both immunocytochemistry and Western blotting, whereas only one of them, BXP-34, gave positive staining in both methods.

Moreover, the functional tests performed on endothelial cells gave us another set of results. Hence, it is impossible to investigate the expression level or function of mdr protein using only one classical method, especially in cells with low mdr expression.

There are several published results indicating that the major problem is insufficient reliability and accuracy of methods used for expression and functional assessment of mdr in different tumor cells [34, 44–46]. On the other hand, some recent publications suggest that there is no simple correlation between mdr expression at mRNA and protein levels and their transporter activities [24], which was also confirmed by our studies with endothelial cells as a cellular model.

Since there are so many problems with proper analysis of mdr expression and functions, we strongly recommend investigating not only the mRNA or protein expression levels but also the mdr activity for the proper assessment of a given mdr protein’s role in biological functions of cells. One of the main reasons for such an approach is that the expression data only roughly reflect the transporter function. Therefore, functional assays may fundamentally affect the conclusion.

## Supporting information

S1 Fig Representative histograms of multidrug resistance protein expression evaluated by flow cytometry methods. White cells—black histogram, isotypic control—red histogram, MDR protein expression—green histogram.
(PDF)

S2 Fig Representative histograms of the multidrug resistance assays’ results. Unstained cells—black histogram, inhibitor treated stained cells—red histogram, stained control cells—green histogram. Multidrug resistance activity factors [%] are shown in each graph.
(PDF)

S1 File Raw data. Raw data obtained for mdr protein expression and function evaluated by flow cytometry methods.
(XLS)
