# Supplementary figures and images for: Expression and activity of multidrug resistance proteins in mature endothelial cells and their precursors: A challenging correlation
Source: PLoS One. 2017 Feb 17;12(2):e0172371. doi: 10.1371/journal.pone.0172371 (PMC5315393; doi:10.1371/journal.pone.0172371)

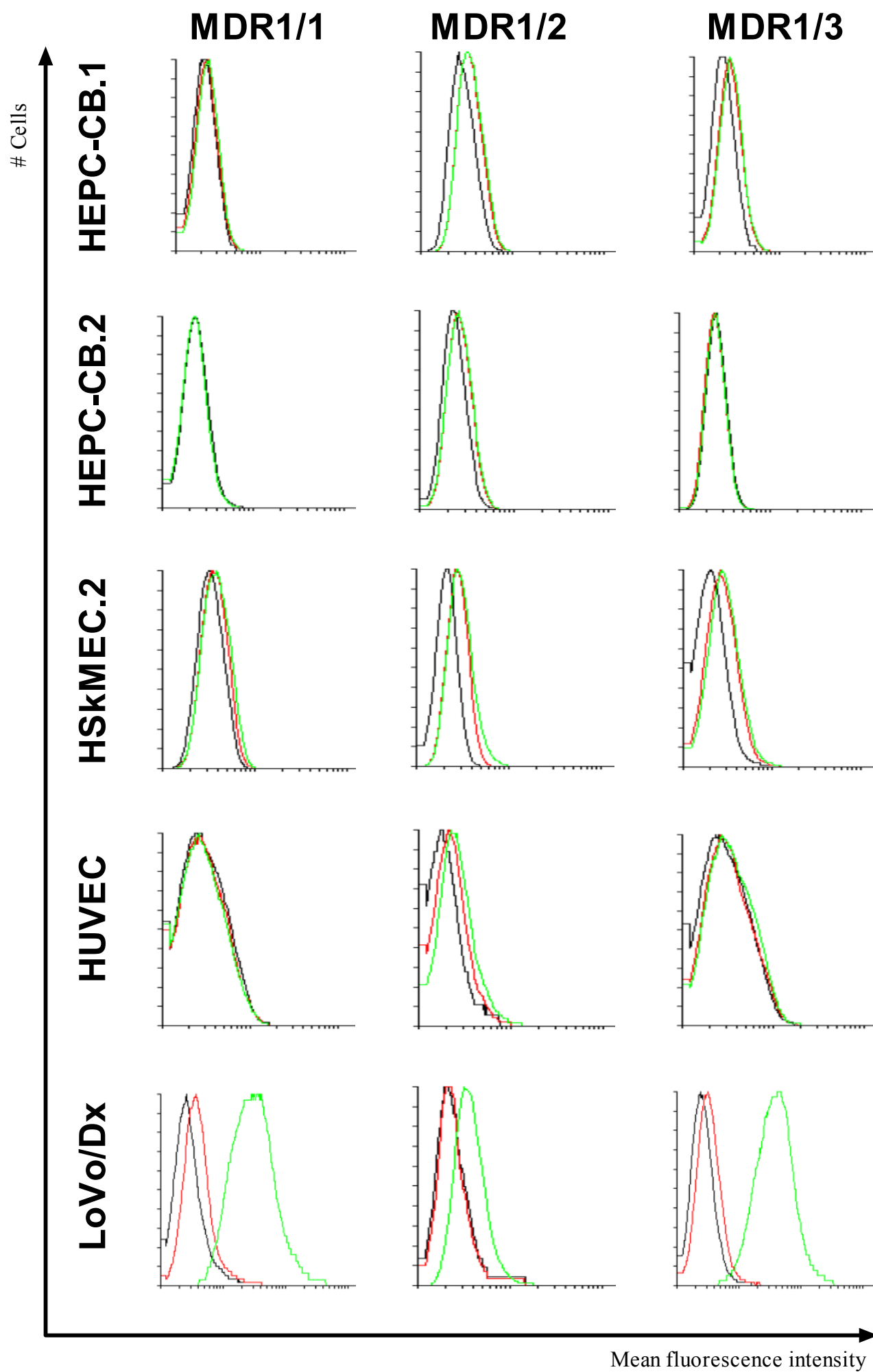

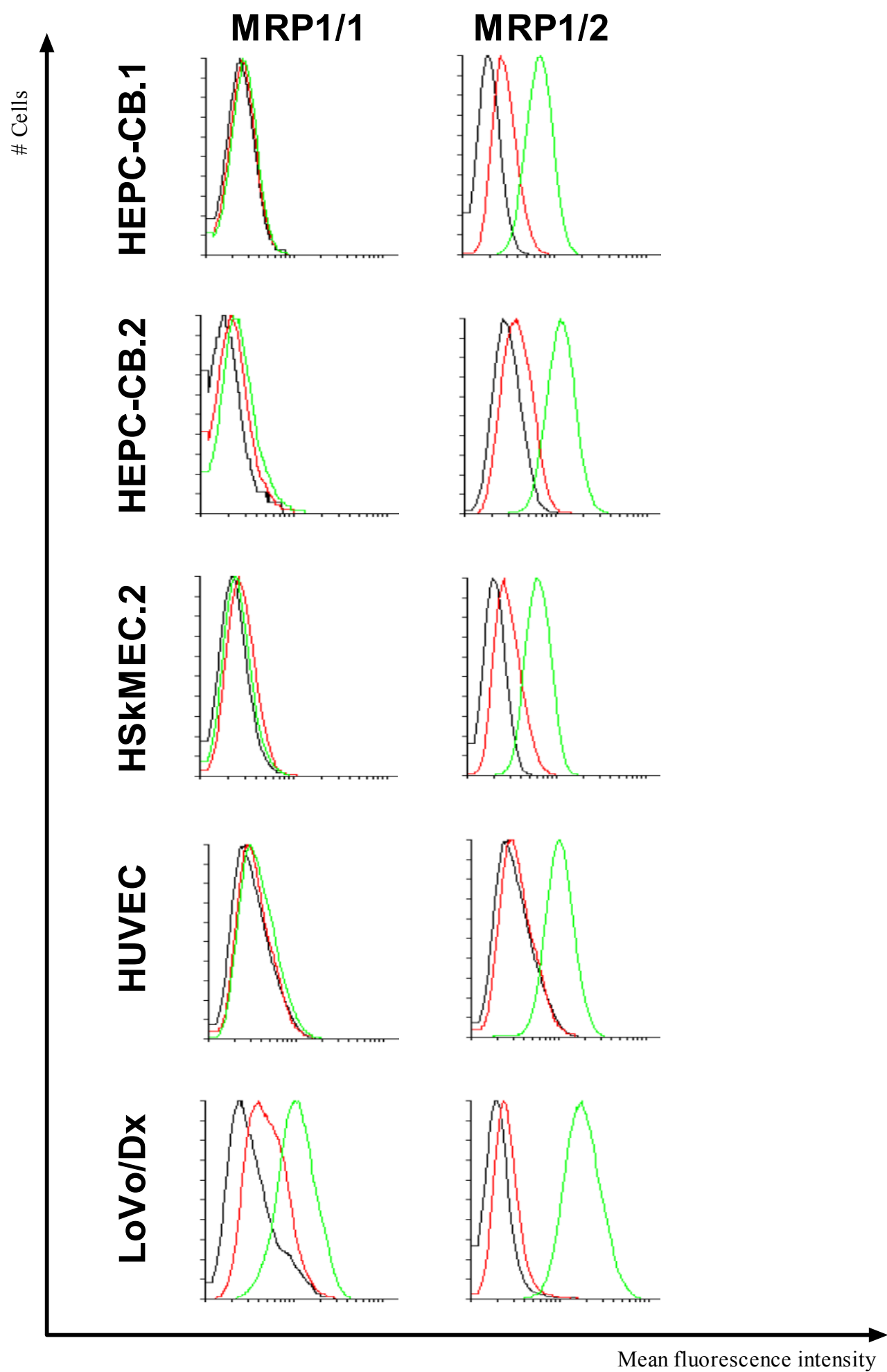

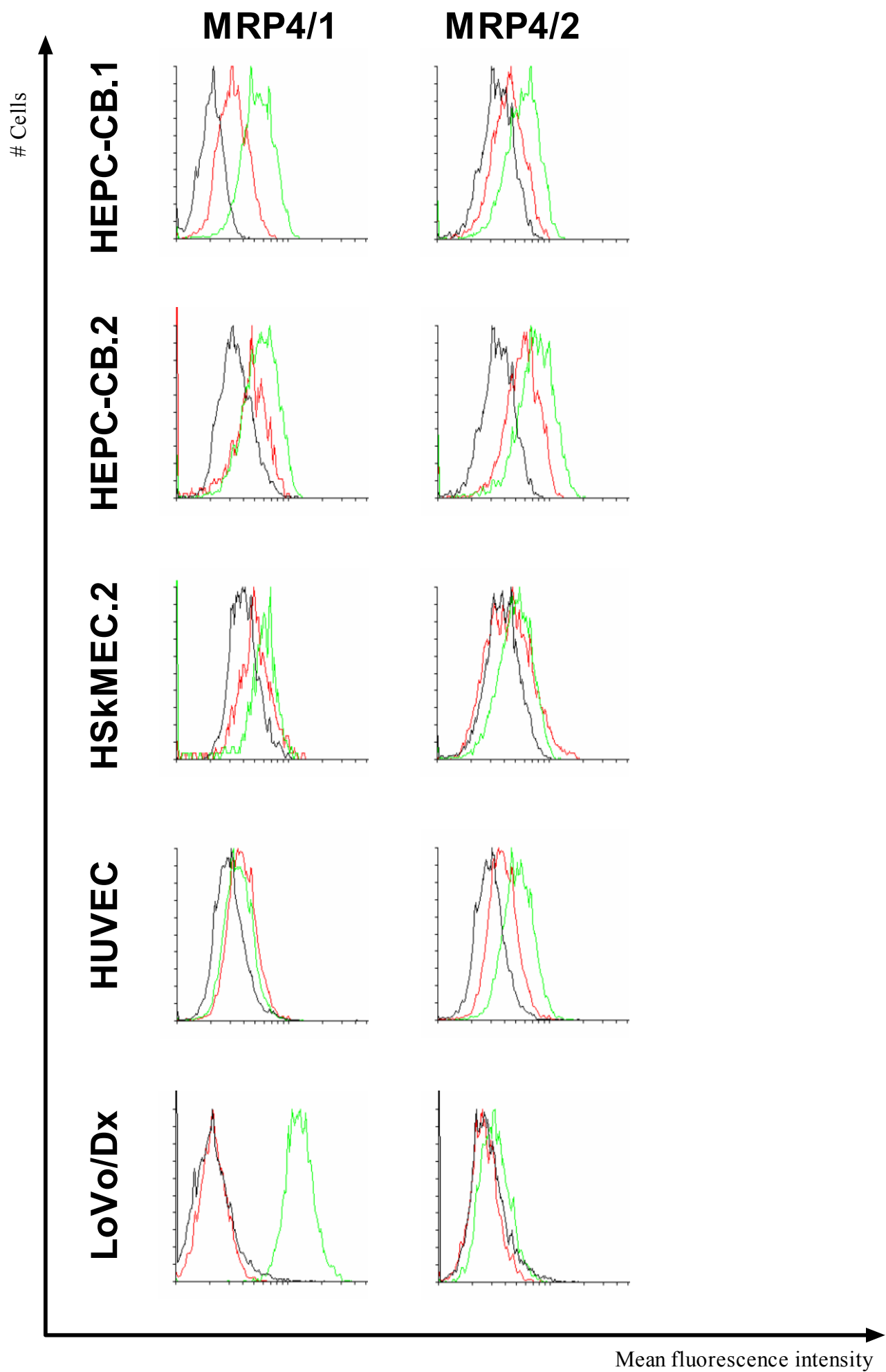

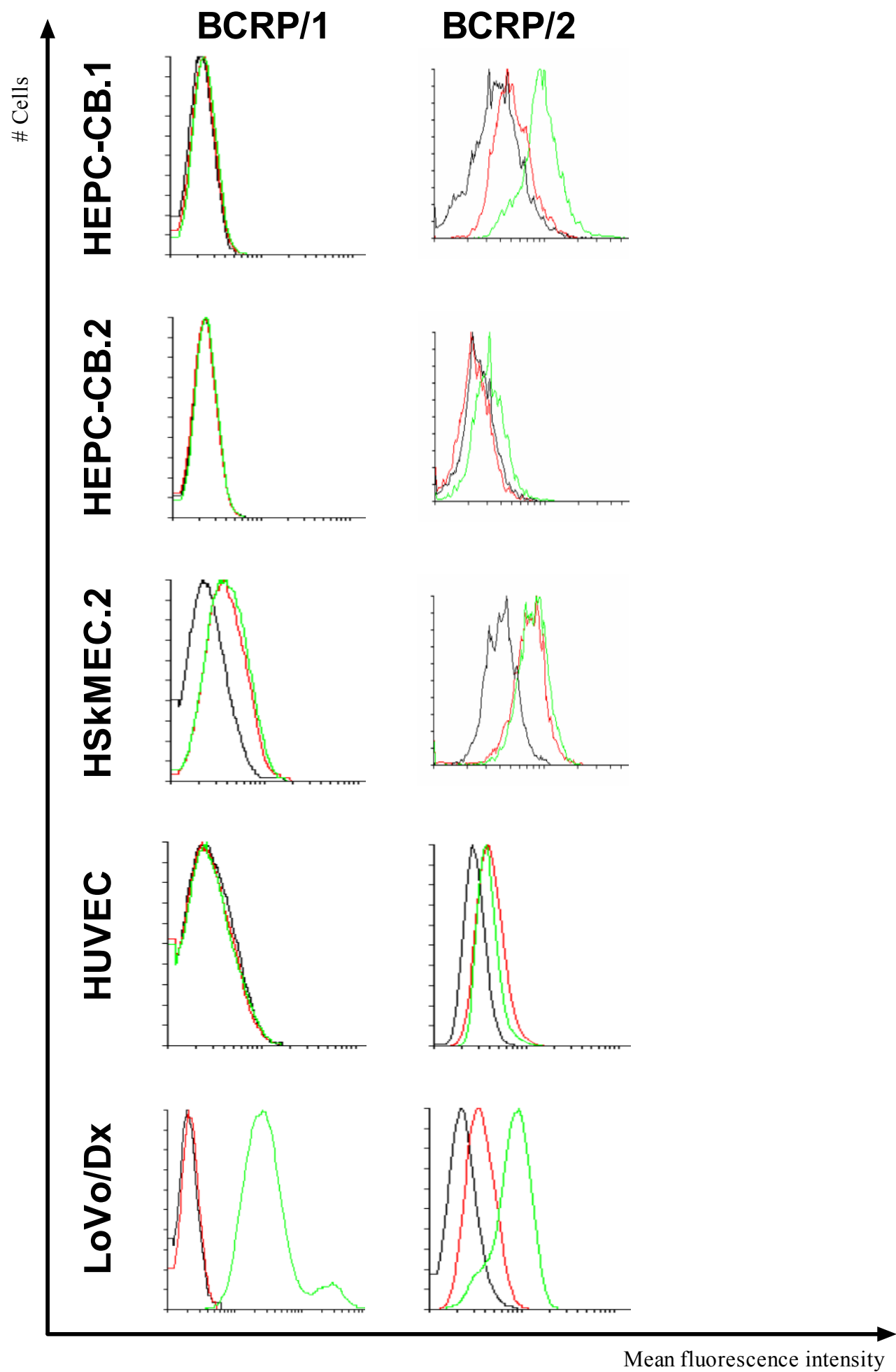

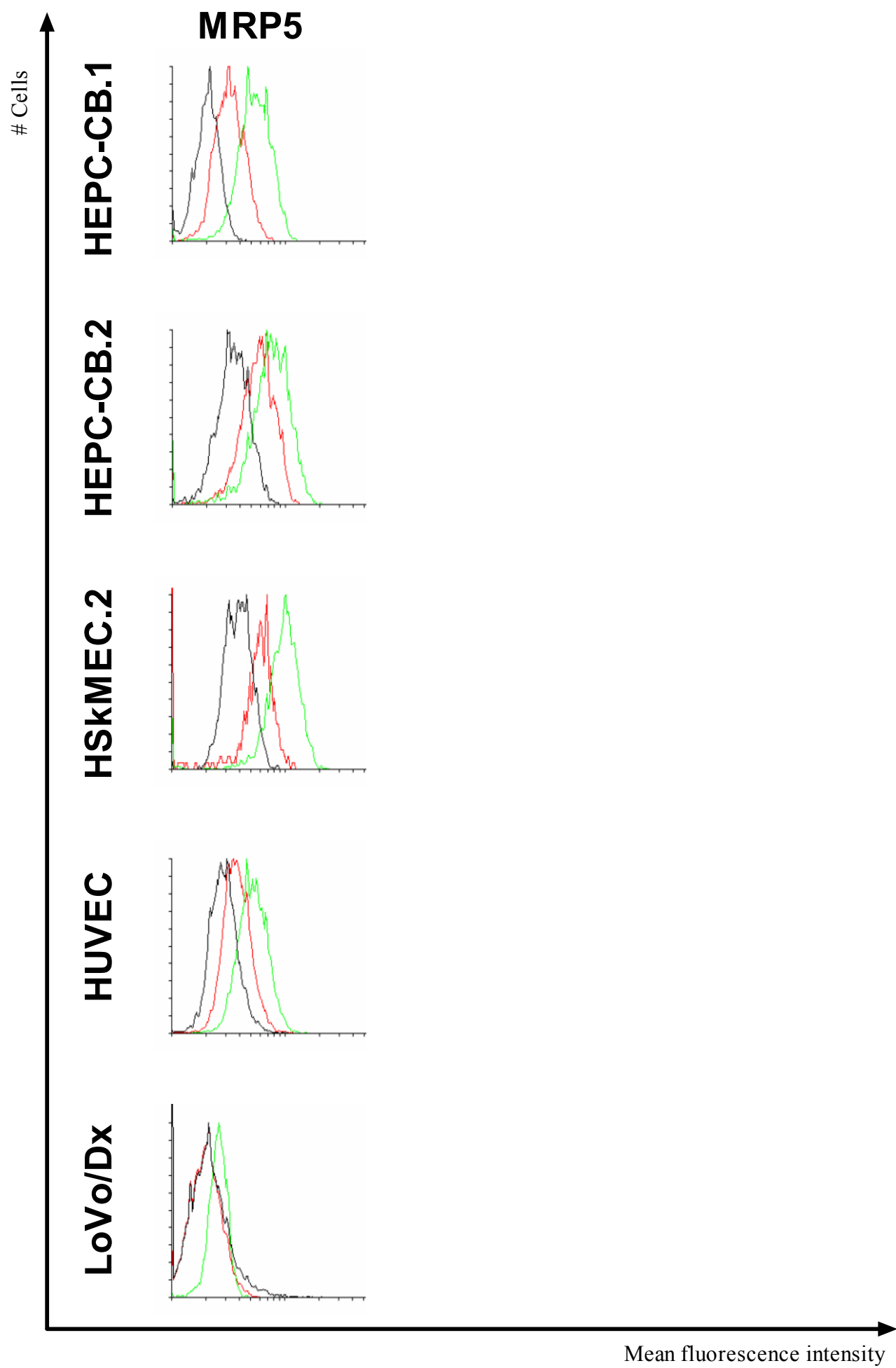

Supplement: S1 Fig — White cells—black histogram, isotypic control—red histogram, MDR protein expression—green histogram. (PDF) [file pone.0172371.s001.pdf]

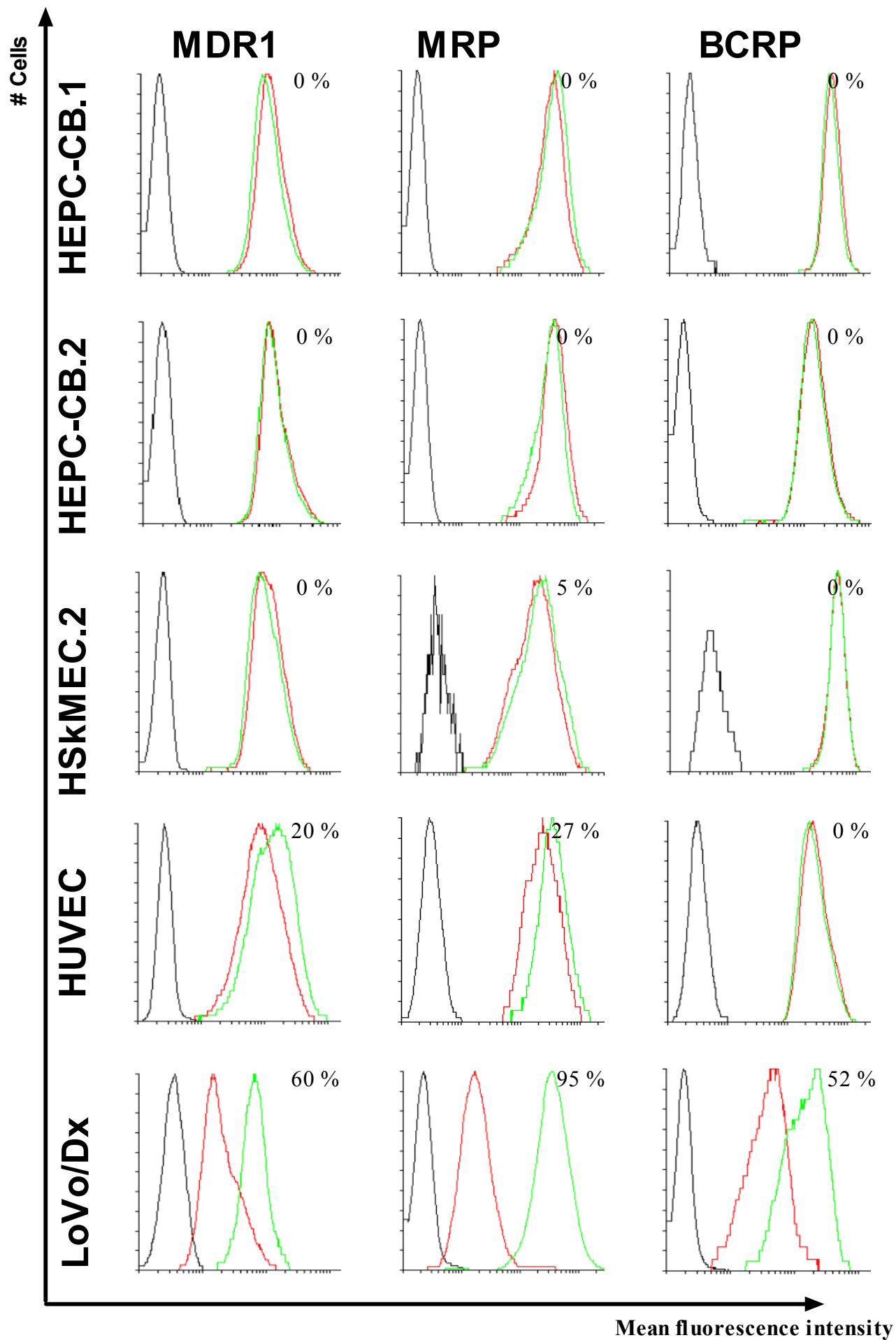

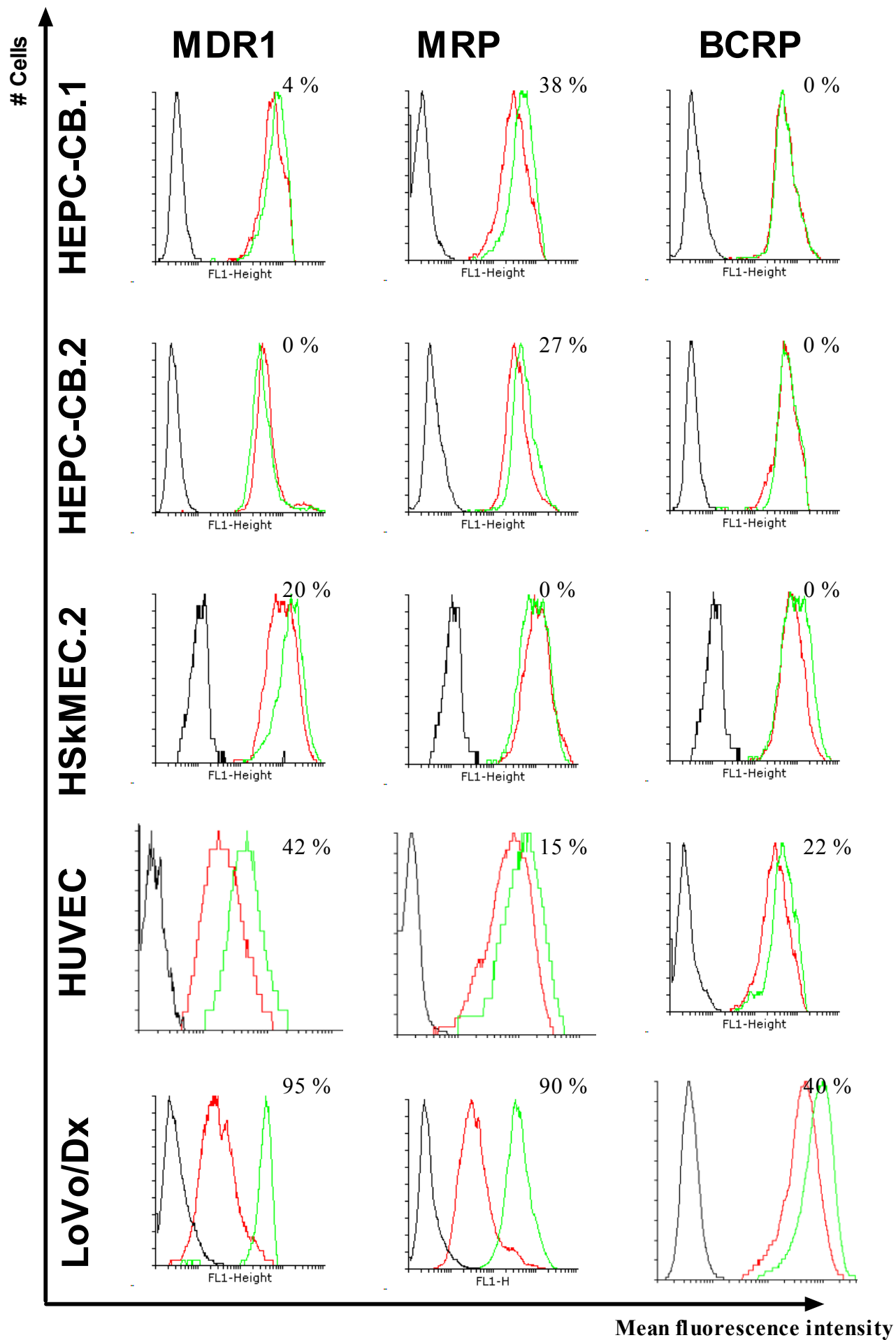

Supplement: S2 Fig — Unstained cells—black histogram, inhibitor treated stained cells—red histogram, stained control cells—green histogram. Multidrug resistance activity factors [%] are shown in each graph. (PDF) [file pone.0172371.s002.pdf]
